# Improving chemical similarity ensemble approach in target prediction

**DOI:** 10.1186/s13321-016-0130-x

**Published:** 2016-04-23

**Authors:** Zhonghua Wang, Lu Liang, Zheng Yin, Jianping Lin

**Affiliations:** State Key Laboratory of Medicinal Chemical Biology and College of Pharmacy, Nankai University, Weijin Road, Tianjin, China; High-Throughput Molecular Drug Discovery Center, Tianjin Joint Academy of Biomedicine and Technology, Tianjin, China

**Keywords:** Fingerprint, Similarity, Off-target effect, Target identification

## Abstract

**Background:**

In silico target prediction of compounds plays an important role in drug discovery. The chemical similarity ensemble approach (SEA) is a promising method, which has been successfully applied in many drug-related studies. There are various models available analogous to SEA, because this approach is based on different types of molecular fingerprints. To investigate the influence of training data selection and the complementarity of different models, several SEA models were constructed and tested.

**Results:**

When we used a test set of 37,138 positive and 42,928 negative ligand-target interactions, among the five tested molecular fingerprint methods, at significance level 0.05, Topological-based model yielded the best precision rate (83.7 %) and ${F_{0.25}{\text{-}}Measure}$ (0.784) while Atom pair-based model yielded the best $F_{0.5}{\text{-}}Measure$ (0.694). By employing an election system to combine the five models, a flexible prediction scheme was achieved with precision range from 71 to 90.6 %, $F_{0.5}{\text{-}}Measure$ range from 0.663 to 0.684 and $F_{0.25}{\text{-}}Measure$ range from 0.696 to 0.817.

**Conclusions:**

The overall effectiveness of all of the five models could be ranked in decreasing order as follows: Atom pair $\approx$ Topological > Morgan > MACCS > Pharmacophore. Combining multiple SEA models, which takes advantages of different models, could be used to improve the success rates of the models. Another possibility of improving the model could be using target-specific classes or more active compounds.

**Electronic supplementary material:**

The online version of this article (doi:10.1186/s13321-016-0130-x) contains supplementary material, which is available to authorized users.

## Background

In recent years, with the increasing cost of drug development and the inconsistent and slow speed of drug approval, predicting new targets for approved drugs has become a popular research area [1–8]. It is well known that drugs interact with multiple targets rather than with a single target (called the off-target effect), and this fact can be beneficial [9] or harmful [10] (known as side effects or toxicity). Drug discovery methods that take advantage of the polypharmacological nature of drugs are becoming more popular [11], because drug discovery starting from approved drugs can benefit from the elimination of many toxicological and pharmacokinetic assessments.

With the ever-increasing public availability of bioactivity data [12], it is possible to construct reliable target-prediction models using statistical or machine learning methods. Paolini et al. [13] identified different types of targets within the human pharmacological interaction network using Bayesian classification models. Using activity data from the ChEMBL17 database, Afzal et al.  [1] evaluated a multi-label multi-class classification model and a single-label multi-class classification model. In 2007, Keiser et al. [5] developed the chemical similarity ensemble approach (SEA), which relates proteins to one another based on the chemical similarity among their bound ligands. Since then, the SEA and SEA-like methods have been successfully applied in new target identification for old drugs [3, 5, 8]/natural products [14], for side-effect prediction [15] and for the prediction of potential anatomical therapeutic indications (ATCs) of approved drugs [16]. Moreover, studies [17] have shown that there is a startling difference between ligand-based and sequence-based approaches, and in most case the ligand-based similarity approach is more informative for pharmacology than the sequence-based approach [4]. Therefore, relating proteins on the basis of the chemical similarity of their ligands, which is motivated by the BLAST theory [18], rather than by their protein sequences, could provide new insights into the relationships between structurally dissimilar but functional related proteins.

An SEA model can be built based on different types of molecular fingerprints. Hert et al. [4] evaluated the performance of several commonly used fingerprints in SEA, and their results showed that ECFP_4 (extended connectivity fingerprint with radius equals 4) yielded the best performance, but the others were comparable. Hence, the chemical similarity criteria of small molecules play key roles in SEA modeling. In this study, to investigate the influence of different fingerprints, training data sets, and activity thresholds on SEA models, we constructed five SEA models based on five fingerprints—Morgan, Atom pair, Topological, MACCS (molecular access system) keys and Pharmacophore—and also a multi-voting SEA model based on the 5 different fingerprint-based SEA models. Finally, we tested the performance of the six SEA models.

## Methods

### Data sets and preparation

**Fig. 1:**
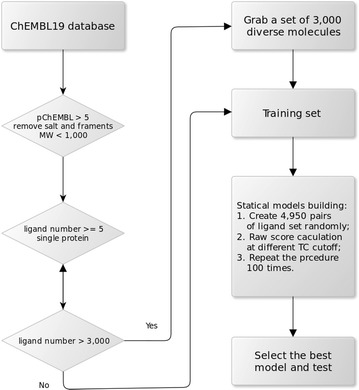
Workflow of SEA. Data workflow and simple procedure of building an SEA model

The ChEMBL database is a good open access data source for drug discovery [12]. In this study, the activity data from ChEMBL19 were used for the training set, whereas the newly reported activity data in ChEMBL20, compared to ChEMBL19, were used as the test set. The following steps were performed to create the training sets. First, as shown in Fig. 1, molecules were curated by removing salt and fragments and by filtering out molecules with MWs (molecule weights) larger than 1000. Second, for target-ligand pairs with multiple activity values, the geometric mean was used. Only targets labeled with SINGLE PROTEIN were used, and targets with fewer than 5 ligands were also excluded; Third, three different activity thresholds (pChEMBL values 5, 6, and 7—the pChEMBL value is a ChEMBL-converted value, which is a negative logarithm of the published activity [19], so 10 μm equals a pChEMBL value of 5) were applied to generate three datasets. Fourth, considering computational efficiency and data balance, although SEA has a robust set size [5], 3000 diverse ligands were picked for targets with ligand set size exceeding 3000. To prepare the test set, the same procedure was applied but with the difference that only one activity threshold (pChEMBL $\ge$ 5) was used. In addition, to test the SEA on a specific protein family, a kinase-specific training set and a test set were created using the same strategy from the kinase activity data of ChEMBL19 and ChEMBL20. Finally, six data sets—training sets with activity thresholds $10$, $1$ and $0.1\ \upmu {\text{m}}$, a test set, a kinase training set and a kinase test set—were generated (see Additional files 1, 2, 3, 4, 5, 6). The data statistics are shown in Table 1.

**Fig. 2:**
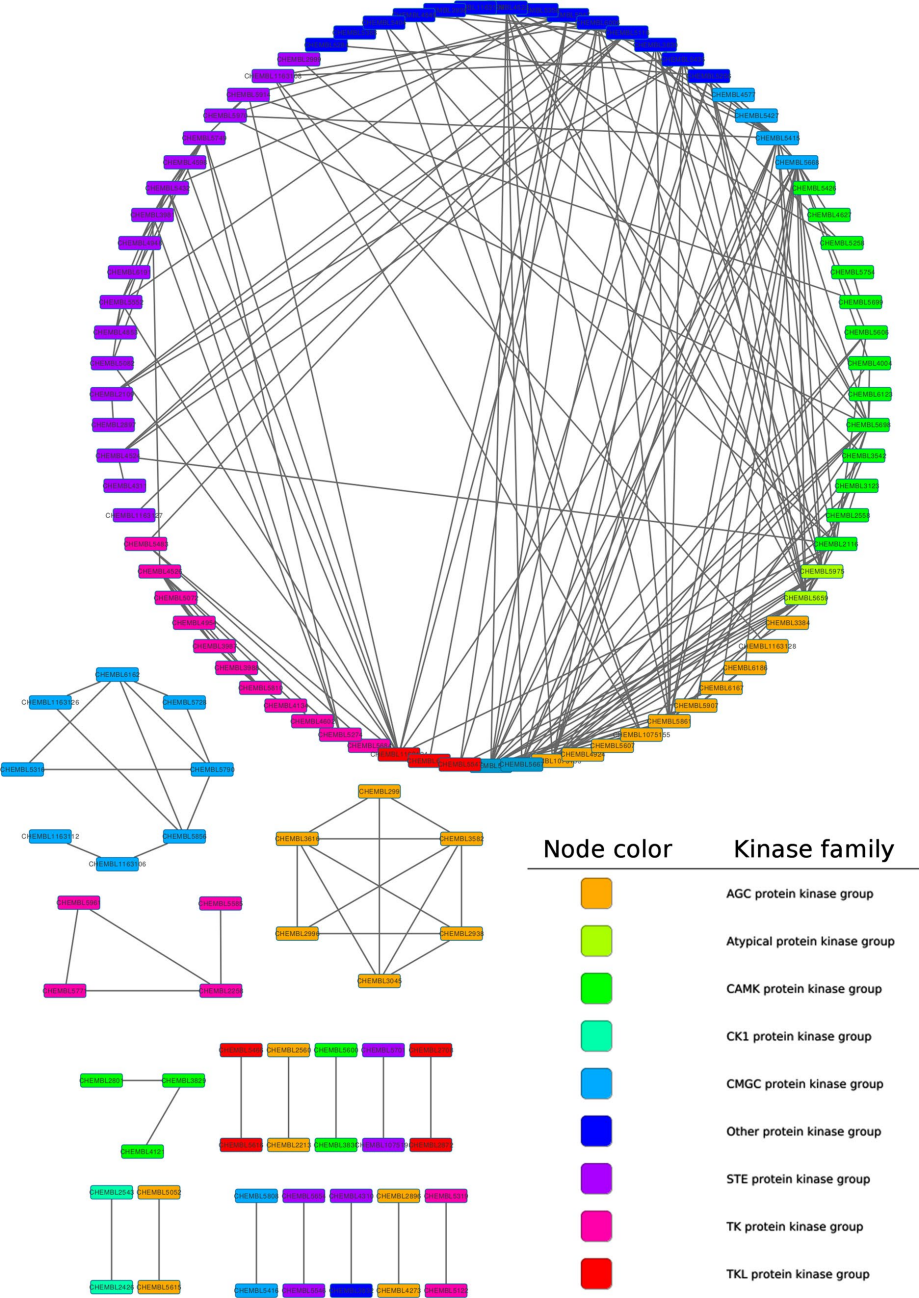
The upper plot illustrates the total number of positive (in *red*) and true positive predictions (in *light blue*) with different vote numbers, and the lower part is the corresponding precision

**Fig. 3:**
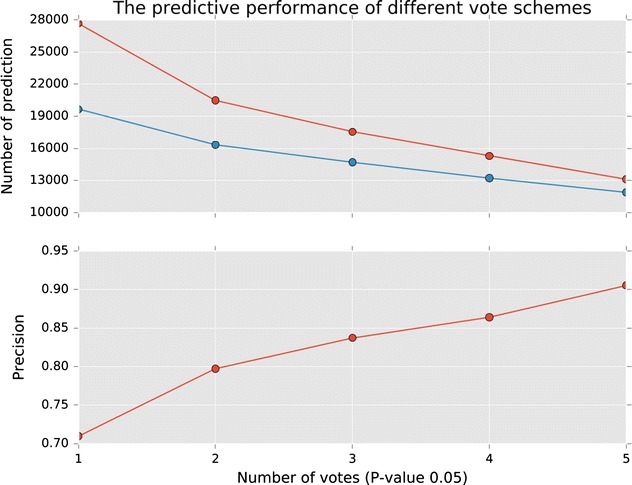
Target relation network for kinase using a kinase-specific SEA model. The *nodes* represent targets, and the linkages indicate significant (P value ${\le} 10^{-80}$) relationships predicted by SEA. The nodes are colored according to 9 kinase subfamily types

### Similarity evaluation and performance validation measures

Only 2D structural similarities were considered in this study. Six different molecular representations were calculated including Morgan (RDKit [20] implementation, similar to the ECFP/FCFP fingerprint [21]), Atom pair fingerprints [22], Topological torsions fingerprints, MACCS keys fingerprints, 2D pharmacophore fingerprints and SHED descriptors [23]. The first five fingerprints are binary vectors that encode the presence or absence of a predefined feature (e.g., a fragment), and the SHED descriptors were calculated based on the information-theoretical concept of Shannon entropy to quantify the variability in a feature-pair distribution [23]. A SHED descriptor is a 10-dimensional array, in which each variable ranges from 0 to 20. The average similarities of the 5 binary fingerprints and SHED descriptors on the active molecules of 2089 ligand sets (of different targets) from the training set were summarized in the (see Additional file 7: Fig. S1).

For binary fingerprint similarity measurements, the Tanimoto coefficient (TC) was used, which is given by Eq.  1:

1 $$\begin{aligned} S_{A, B} = \frac{c}{ a + b + c}, \end{aligned}$$

where S represents the coefficient, a and b are the on bits of A and B, and c is common to both bits. Moreover for SHED descriptors, the similarity of A and B is given by Eq. 2:

2 $$\begin{aligned} S_{A, B} = 1 - \frac{DIST(A, B)}{20\sqrt{10}}, \end{aligned}$$

where *DIST*(*A*, *B*) denotes the Euclidean distance between A and B.

The performances of each model were evaluated with respect to accuracy, precision, sensitivity, specificity and $F_\beta{\text{-}}Measure$ as shown in the Eqs. (3–7). The $F_\beta{\text{-}}Measure$ is the harmonic mean of precision and sensitivity. It combines precision and sensitivity in a single metric. More specifically, the $F_\beta{\text{-}}Measure$ is a weighted harmonic mean of precision and sensitivity in which $\beta$ measures the effectiveness of retrieval with respect to a user who attaches $\beta$ times as much importance to sensitivity as precision. For example, the $F_{0.5}{\text{-}}Measure$ and $F_{0.25}{\text{-}}Measure$ weights precision two and four times more than sensitivity, respectively. In this study, due to the incomplete experimental evidence of the relationship of all ligand-target pairs in both test and training data set, the multi-label classification problem, that a ligand may be active against more than one target, was convert to binary classification. Thus, the false positive rate obtained is underrated, which will be discussed in the result section. Under this circumstances, precision is more important than sensitivity, therefore, two variations of $F_\beta{\text{-}}Measure$, $F_{0.5}{\text{-}}Measure$ and $F_{0.25}{\text{-}}Measure$ together with precision, were mainly used to examine and discuss the results of different models.

3 $$\begin{aligned} Accuracy= \frac{TP + TN}{TP + FP + TN + FN} \end{aligned}$$

4 $$\begin{aligned} Precision= \frac{TP}{TP + FP} \end{aligned}$$

5 $$\begin{aligned} Sensitivity= \frac{TP}{TP + FN} \end{aligned}$$

6 $$\begin{aligned} Specificity= \frac{TN}{FP + TN} \end{aligned}$$

7 $$\begin{aligned} F_{\beta}{\text{-}}Measure= (1 + \beta ^ 2) \times \frac{Precision \times Sensitivity}{\beta ^{2} \times Precision + Sensitivity} \end{aligned}$$

where TP, FP, TN and FN denote true positive, false positive, true negative and false negative respectively.

### SEA model implementation

The procedures for building SEA models were derived from a reference [5], with minor changes. Here, a brief summary is provided. The chemical similarity of two sets of ligands can be accessed by the sum of the chemical similarities between each pair of ligands. However, this process will render the value very sensitive to the size of the data, to noise and to false positive data. To minimize the influence of noise, the original SEA [5] method introduced the Raw Score (RS) (Eqs. 8, 9), which was defined as the sum of the ligand-pair TCs over all of the pairs with $TC \ge TS$ (Tanimoto threshold). Then, RS was converted to a Z-score and P value (see eqs.10–14), which were used to indicate the significance of the RS. In addition, TS was determined by the best fitness of EVD (extreme value distribution) using the chi-square test, indicating that only significant similarities were considered contributions to set-set similarity. This work followed Keiser et al.’s [5] procedures to fit TS, with RS calculated for all TC thresholds from 0.00 to 0.99 with a step size of 0.01. As described in Fig.  1, after data curation, the background data sets were randomly created with set sizes ranging from 10 to 1000 and an interval step of 10, which results in 4950 pairs of molecular data set. Then, pairwise RS of data sets were calculated, this RS calculation procedures is described in detail using its pseudo code (illustrated in Algorithm 1). This procedure was repeated 100 times. More details of the procedure can be found in the original work [5].

8 $$\begin{aligned} Rawscore(A, B) = \sum _{i}\sum _{j}SIM(A_i, B_j), \end{aligned}$$

where

9 $$\begin{aligned} SIM(a, b)= {\left\{ \begin{array}{ll} TC(a, b) {\text{ if }} TC(a, b) \ge TS; \\ 0 {\text{ if }} TC(a, b) < TS. \end{array}\right. } \end{aligned}$$

10 $$\begin{aligned} Z-score= \frac{Rawscore(A, B)-F_{mean}(s)}{F_{sd}(s)}, \end{aligned}$$

where *s* is the product of set A and B, $F_{mean}$ and $F_{sd}$ are:

11 $$\begin{aligned} F_{mean}(x)= \mu x; \end{aligned}$$

12 $$\begin{aligned} F_{sd}(x)= \phi x ^{\eta }. \end{aligned}$$

Functions $F_{mean}$ and $F_{sd}$ were used to calculate the expected raw score mean and standard deviation, and the parameters $\mu$, $\phi$ and $\eta$ were determined by fitting the random background statistical model (see the Additional file 7: Fig. S2 and S3). Considering the fact that for $z \ge 28$, computing $e^z$ exceeds the numerical precision of most programming languages, therefore a Taylor expansion is employed instead [5]. Then, the P value of a Z-score (*z*) was calculated:

13 $$\begin{aligned} P{\text{-}}value(z)= {\left\{ \begin{array}{ll} 1 - e^{x(z)}\quad {\text{ if }} z \le 28;\\ -x(z) - \frac{x(z)^2}{2} - \frac{x(z)^3}{6}) \quad {\text{ if }} z > 28. \end{array}\right. } \end{aligned}$$

where

14 $$x(z) = -e^{-\frac{-z\pi }{\sqrt{6} - 0.577215665}}.$$

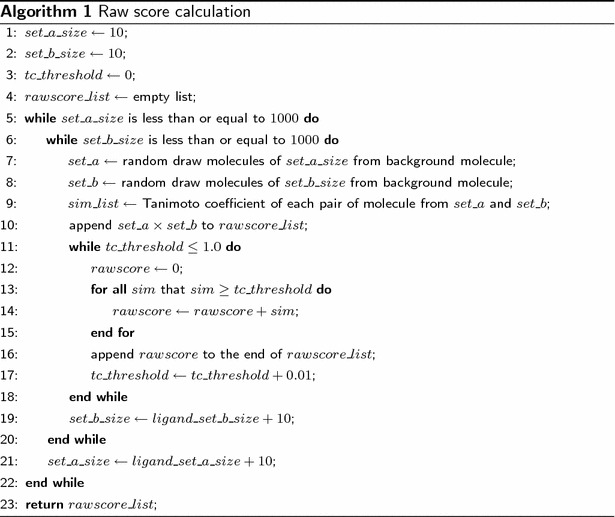


## Results and discussion

### Activity threshold

Generally, $10\ \upmu {\text{m}}$ has been used as activity cutoff in many works [24, 25]. However, to investigate the influence of different activity thresholds, three SEA models were constructed with activity thresholds of $10$, $1$ and $0.1\ \upmu {\text{m}}$, respectively. All the three models were built based on Morgan fingerprint. The result, as shown in Table 2, showed that, at the significance level of P value $$\le \ $$0.05, the model with a threshold of $0.1\ \upmu {\text{m}}$ yielded the best precision of 95.8 % and specificity of 99.7 %, but a very low sensitivity (true positive rate or recall) of 7.2 %; however, the model with a threshold of $10\ \upmu {\text{m}}$ yielded the best accuracy (67.6 %), sensitivity (38.2 %), and $F_\beta{\text{-}}Measure$ ($F_{0.5}{\text{-}}Measure = 0.57$, $F_{0.25}{\text{-}}Measure = 0.772$). And the performance of the model with $1\ \upmu {\text{m}}$ as threshold is in between the above two models. This result should not come as surprise because a higher activity threshold indicates a higher quality of the training set, as well as a smaller size of the set. It must be point that, of the 1190 * 26,489 ligand-target pairs in test set, Morgan model with threshold $10\ \upmu {\text{m}}$ gave 65,772 pair of positive predictions (P value $\le$0.05), and most of these predictions haven’t been proved by experiment. Here we took a conservative estimate of the real result that the false positive rate was underestimated. Therefore, in the following sections, $F_{0.5}{\text{-}}Measure$ and $F_{0.25}{\text{-}}Measure$ were used as the measure. On the other side, at the significance level of P-value ≤0.01, the precision, accuracy $F_{0.5}{\text{-}}Measure$ and $F_{0.25}{\text{-}}Measure$ of the model with a threshold of $10\ \upmu {\text{m}}$ reached at 91.6, 67.9 %, 0.684 and 0.883 respectively but with the expense of reduction of sensitivity (33.9 %). Thus, in practice, it depends on the researchers to decide which model to use, according to the actual situation, need broader alternatives of ligand-target interaction pair for a few of potential molecule or a higher predictive accuracy rate for high-throughput target identification for a large molecule set. For consistency, hereafter in this paper, unless otherwise specified, the models were built using the training data set, filtered with an activity threshold of $10\ \upmu {\text{m}}$.

**Table 1:** Statistics of the training and test sets

Data set	Target	Molecule	Ligand-target pair	Active
Training set (5)	2,809	393,090	666,313	All
Training set (6)	2,297	294,877	407,296	All
Training set (7)	1,711	179,710	246,651	All
Kinase training set (5)	429	42,164	101,502	All
Test set	1190	26,498	80,066	37,138
Kinase test	259	2,225	3010	2,192

The size of 4 training data sets and 2 test sets. Numbers in brackets denote activity thresholds

**Table 2:** Predictive results of SEA models with different activity thresholds (P value ≤ 0.05)

Threshold (μm)	TS	Accuracy	Precision	Sensitivity	Specificity	$F_{0.5}{\text{-}}Measure$	$F_{0.25}{\text{-}}Measure$
0.1	0.69	0.568	0.958	0.072	0.997	0.278	0.557
1	0.69	0.592	0.94	0.129	0.993	0.417	0.687
10	0.62	0.676	0.826	0.382	0.93	0.67	0.772

### Fuzzy representation of compounds

The two-dimensional Pharmacophore fingerprint implemented in the RDKit [20] package was employed to investigate the influence of the “fuzziness” of the representation of compound structures in the SEA model. Details of the definition can be found in the RDKit online document (http://rdkit.org/docs/RDKit_Book.html). The different levels of fuzziness were controlled by the number of points of the pharmacophore and the shapes of the bins. The fingerprint definition from Gobbi’s work [26], which is also implemented in RDKit, was used in this study. Table 3 demonstrates the target prediction performances of 3 types of pharmacophore definitions. With the same 2 to 3 points in a pharmacophore, the comparison between differently shaped bins showed that rougher bin selection, indicating a fuzzier fingerprint, yielded higher sensitivity (43.6 vs. 42 %) but lower accuracy rate (64.2 vs. 66.7 %), precision (67 vs. 75.2 %), $F_{0.5}{\text{-}}Measure$ (0.61 vs. 0.65) and $F_{0.25}{\text{-}}Measure$ (0.657 vs. 0.719). However, an “extremely fuzzy” fingerprint with only 2 points in a pharmacophore was not sufficiently informative to build an SEA model because it yielded a poor precision rate of 47.9 %, which indicates the false positive rate is more than 50 %. Pharmacophore-based fingerprints are a type of flexible molecular representation because the definition of the pharmacophore and the shape of the bin can vary, resulting in different levels of fuzziness. Fuzzy pharmacophores can also be used to identify compounds with similar pharmacological functions but structural differences [27, 28]. The results in this section indicated that the fuzziness of the pharmacophore impacted the performance of the SEA greatly, and a well-designed pharmacophore scheme might improve the performance significantly. In the following sections, pharmacophore fingerprint-based SEA was built with point numbers of 2 and 3, and bin shapes (2,3), (3,4), (4,5), (5,6), (6,7), and (7,20).

**Table 3:** Predictive results of SEA models with different pharmacophore representations of compounds in fingerprints

Points of pharmacophore	Bin shape	Accuracy	Precision	Sensitivity	Specificity	$F_{0.5}{\text{-}}Measure$	$F_{0.25}{\text{-}}Measure$
2	(0,2), (2,5), (5,8)	0.513	0.479	0.567	0.466	0.494	0.483
2, 3	(0,2), (2,5), (5,8)	0.642	0.678	0.436	0.821	0.61	0.657
2, 3	(2, 3), (3, 4), (4, 5), (5, 6), (6, 7), (7, 20)	0.667	0.752	0.42	0.88	0.65	0.719

**Table 4:** At significance level 0.05, the test result of different SEA models. The numbers after “Multi-voting” denote each voting scheme, e.g. Mult-voting (3) is a 3-vote scheme

	Accuracy	Precision	Sensitivity	Specificity	$F_{0.5}{\text{-}}Measure$	$F_{0.25}{\text{-}}Measure$
Atom pair	0.692	0.817	0.432	0.916	0.694	0.777
MACCS	0.682	0.802	0.417	0.911	0.677	0.76
Morgan	0.676	0.826	0.382	0.93	0.67	0.773
Topological	0.682	0.837	0.39	0.934	0.681	0.784
Pharmacophore	0.667	0.752	0.42	0.88	0.65	0.719
Multi-voting (1)	0.681	0.71	0.529	0.813	0.664	0.696
Multi-voting (2)	0.688	0.797	0.44	0.903	0.686	0.761
Multi-voting (3)	0.684	0.837	0.396	0.933	0.684	0.786
Multi-voting (4)	0.675	0.864	0.356	0.952	0.672	0.797
Multi-voting (5)	0.669	0.906	0.32	0.971	0.663	0.817

**Table 5:** The number of overlaps of true positive predictions of each SEA model

	Atom pair	MACCS	Morgan	Topological	Pharmacophore
Atom pair	16,044	13,853	13,335	13,805	13,600
MACCS	13,853	15,478	13,010	13,191	13,084
Morgan	13,335	13,010	14,176	13,282	12,902
Topological	13,805	13,191	13,282	14,467	12,814
Pharmacophore	13,600	13,084	12,902	12,814	15,594

### SHED descriptors and Euclidean distance

We also tested the probability of SHED in building an SEA model. SHED is a pharmacophore-based descriptor schema including 4 pharmacophore definitions—hydrophobic, donor, acceptor and aromatic—as well as 10 pairwise descriptors. As stated in the Methods section, Euclidean distance together with a normalized Eq. (2), was used as a similarity criterion. Unlike with EVD, the Z-scores achieved from SHED followed a Gaussian distributions more closely. Although SHED has been successfully used in some works [29, 30], the test results in this study showed that this type of schema is not proper for SEA models with poor precision (45.4 %) as well as $F_{0.5}{\text{-}}Measure$ (0.481) and $F_{0.25}{\text{-}}Measure$ (0.462), indicating that SHED, with 10 dimensional arrays, is not sufficiently informative to build an accurate SEA model.

### SEA with different types of fingerprints

To analyze the predictive power of different fingerprints in SEA models, in addition to Morgan and pharmacophore models, another 3 SEA models were also built, including Atom pair, MACCS keys and Topological models. Table 4 shows the test results of 5 fingerprint-based SEA models. The prediction precision rates of the five fingerprint-based SEA models ranged from 75.2 to 83.7 % (at a P value $\le$0.05) or from 85.6 to 92.1 % (at a P value $\le$ 0.01). More specifically, at significance level 0.05, The Topological model yielded the best precision rate (83.7 %) and $F_{0.25}{\text{-}}Measure$ (0.784) while The Atom pair model yielded the best $F_{0.5}{\text{-}}Measure$ (0.694). Therefore, the overall effectiveness of all of the models could be ranked in decreasing order as follows: Atom pair $\approx$ Topological > Morgan > MACCS > Pharmacophore. However, as can be observed from Table 4, in general, all the five models are comparable which consisted with previous work [4].

### Multiple-voting SEA model

Kogej et al.’s [31] work demonstrated that much overlap was observed in selecting compounds using different fingerprints, and the combination of different fingerprints yielded better performance [31]. Therefore, it was worthwhile to determine whether combining multiple SEA models could improve the predictive power. First, we calculated the overlaps of the number of true positive predictions of different fingerprint-based SEA models. Table 5 shows that most of the predictions of different models overlapped with each other. Taking Atom pair-based model as an example, of the 15,944 true positive prediction, only 736 predictions overlapped with none of the predictions from other models. This finding was consistent with Kogej et al.’s work. Then, we constructed a multi-voting SEA model, as described in the following. To combine the 5 models, an election system was built by employing the P-value of each model as a vote. For example, if we took 3 votes into consideration (3-vote scheme), a ligand-target pair was significant only if there were more than three P-values less than the P-value cutoff from the five SEA models. The test results of the 1 to 5-vote SEA models are also included in Table 4. As expected, it can be found that precision increase with the vote cutoff of the model. Figure 2 presents the number of positive prediction, true positive prediction and the accuracy rates of different vote schemes at significance level 0.05. The 1-vote scheme yielded 27,676 predictions, of which 19,644 were correct, and this number was more than half of the test set. However, the precision rate was relatively low (71 %). In contrast, the 5-vote scheme yielded a high precision of 90.6 % but a relatively small number of positive predictions at 13,122 (11,882 were true positive). Moreover, with a significance level of 0.01, the 5-vote scheme yielded a high accuracy of 94.1 % (see the Additional file 7: Fig. S4). Our results indicated that combining different fingerprints did improve the predictive performance of the SEA model. Because different fingerprints take charge of different aspects and features of a compound, the multi-voting SEA could be very robust (using a 1-vote scheme) for predicting target-ligand pairs and also accurate in its results (using the 5-vote scheme).

### Kinase specific model

The Target class-specific model, by removing unrelated protein families or noise information, should improve the predictive performance. To confirm this assumption, a kinase-specific SEA model was constructed using a kinase training set based on Morgan fingerprint. When running on the kinase test set (2,192 positives, 818 negatives), at significance level 0.05, the kinase-specific SEA model outperform Morgan-SEA-5 in precision 100 vs. 94.8 %, but Morgan-SEA-5 model gave better $F_{0.5}{\text{-}}Measure$ (0.667 vs. 0.326) and $F_{0.25}{\text{-}}Measure$ (0.843 vs. 0.621) result. Our results indicated that a target class-specific SEA model could improve the prediction precision rate, all positive prediction were correct in this case. Therefore, a kinase-specific SEA model is useful and reliable (due to its high prediction accuracy) for capturing target relationships within the kinase families. As stated above, chemical similarity of the targets may not consist with their sequence similarity. For enzyme activity classes, many targets were pharmacologically similar, with the higher ligands chemical similarity, but sequence dissimilar [5]. Research has also shown that linkage between two targets determined by chemical structural similarity rather than protein sequence might be more useful for drug discovery [4, 32]. Figure 3 shows a target relation network created using the kinase-specific SEA model. For clarity of the graphic illustration, only the most significant predictions are shown in the network (P value ≤$10^{-80}$). Despite the connection inside the subfamily of kinase, more than half (105 of 202) of the connections were across kinase subfamilies. For example, serine/threonine-protein kinase PAK7 and AMP-activated protein kinase alpha-2 subunit share 374 active compounds, and 16 of them are drugs; therefore there is a linkage between these two targets, although they are biologically unrelated (belonging to the STE protein kinase group and the CAMK protein kinase group, respectively).

## Conclusion

In this work, we tested different aspects of SEA models, with the purpose of improving the accuracy rate of an SEA, indicating the activity threshold selection and the use of class-specific sets. The results showed that using stricter (activity cutoffs of 1 or 0.1 μm) and more specific training data could improve the prediction accuracy rate of the SEA model but at the price of a smaller number of correct, positive predictions, indicating a higher false negative rate. To investigate the fuzzy nature of fingerprints, 3 pharmacophore fingerprint-based SEA models were constructed and the comparison indicated that fuzzy fingerprints can yield larger numbers of predictions with overly rough representation, which could lead to very low accuracy rates or even an impractical model. The comparison results of five different models showed that the Topological fingerprint-based SEA model outperformed the other models with the highest precision rate, and the Atom pair-based fingerprint yielded the greatest number of correct, positive predictions. The overall effectiveness of all of the models could be ranked in decreasing order as follows: Atom pair $\approx$ Topological> Morgan> MACCS> Pharmacophore. Although most of the predictions of each model were overlapped, the multi-voting model showed that combining multiple SEA models is a promising method for target prediction. With a tunable vote number, the multi-voting scheme can be flexible in its results, with either a high quality of prediction or a greater number of potential alternatives. It should be noted that the test results in this paper were optimistic because the test set used consisted of newly published data; thus, there were a great number of predictions that could not be proved for now and were not considered in the test results. Target-specific SEA could also improve the prediction accuracy.

An inherent assumption that molecules with similar structures tend to have similar responses to a target underlays SEA method. Thus, the challenge of improving SEA seems to be the same as “the traditional” ligand-based drug discovery methods, such as Quantitative Structure-Activity Relationship or Virtual Screening. These methods suffered from the problem of the activity cliff, which is defined as pairs of structurally similar molecules with large differences in potency [33, 34]. Fingerprints capable of distinguishing these compounds [28] could be used to improve SEA models.

## Additional files

10.1186/s13321-016-0130-x Training data set with activity cutoff 10 μm.
10.1186/s13321-016-0130-x Training data set with activity cutoff 1 μm.
10.1186/s13321-016-0130-x Training data set with activity cutoff 0.1 μm.
10.1186/s13321-016-0130-x Kinase specific training data set with activity cutoff 10 μm.
10.1186/s13321-016-0130-x Test data set.
10.1186/s13321-016-0130-x Kinase test data set.
10.1186/s13321-016-0130-x **Figure S1** The average similarity of five fingerprints (Atom pair, Morgan, MACCS, Topological and Pharmacophore, which are implemented in RDKit package [http://rdkit.org/].) and SHED descriptor. The similarity criteria for SHED is the normalized Euclidean distance (see the main manuscript) and for the other five fingerprints are Tanimoto coefficient. **Figure S2** Statistical model fits for Morgan based SEA on the random background data set create from ChEMBL 19. **Figure S3** Z-score distribution (Morgan fingerprint) of the random background data set created from ChEMBL 19 database.   **Figure S4** ​The predictive performance of different vote schemes with significant level P-value ≤ 0.01. The upper plot illustrates the total number of positive (in red) and true positive prediction (in blue), and the lower plot is the corresponding precision.

## Abbreviations

AUC: area under receiver operating characteristic curve
ECFP_4: extended connectivity fingerprint with radius equals 4
EVD: extreme value distribution
MACCS: molecular access system
MW: molecular weight
RS: raw Score
SEA: similarity ensemble approach
SHED: Shannon entropy descriptors
TC: tanimoto coefficient
TS: tanimoto threshold
