# Supplementary material for: Improving chemical similarity ensemble approach in target prediction
Source: J Cheminform. 2016 Apr 23;8:20. doi: 10.1186/s13321-016-0130-x (PMC4842302; doi:10.1186/s13321-016-0130-x)
Supplement: Supplementary file 6 — 10.1186/s13321-016-0130-x Kinase test data set. [file 13321_2016_130_MOESM6_ESM.pdf]

# Additional File

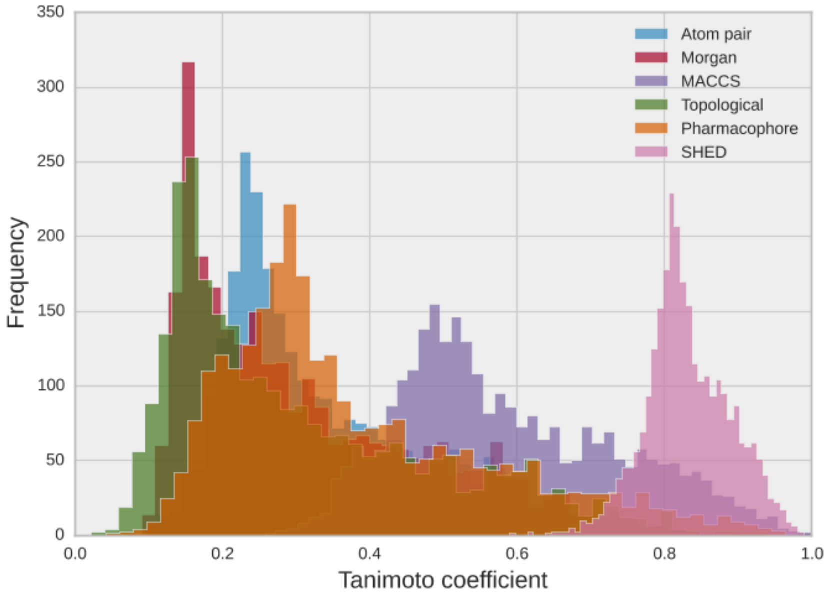


**Figure 1.** The average similarity of five fingerprints (Atom pair, Morgan, MACCS, Topological and Pharmacophore, which are implemented in RDKit package [http://rdkit.org/].) and SHED descriptor. The similarity criteria for SHED is the normalized Euclidean distance (see the main manuscript) and for the other five fingerprints are Tanimoto coefficient.

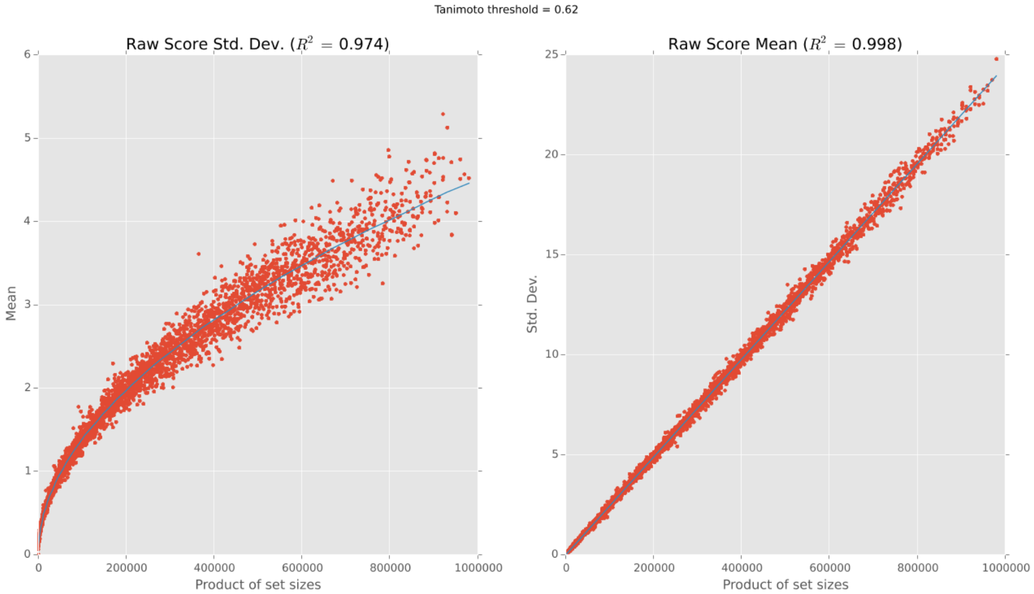


**Figure 2.** Statistical model fits for Morgan based SEA on the random background data set create from ChEMBL 19.

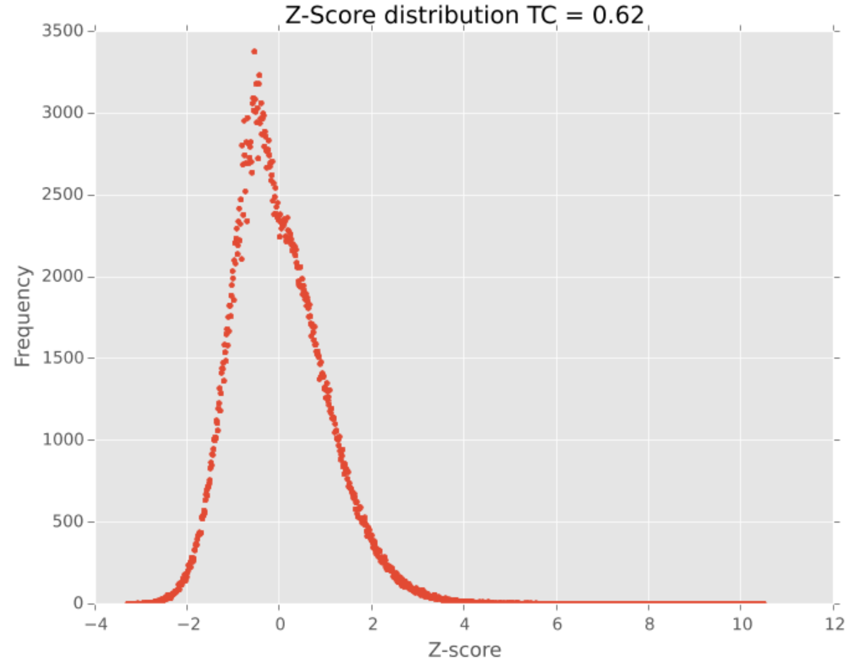


**Figure 3.** Z-score distribution (Morgan fingerprint) of the random background data set created from ChEMBL 19 database.

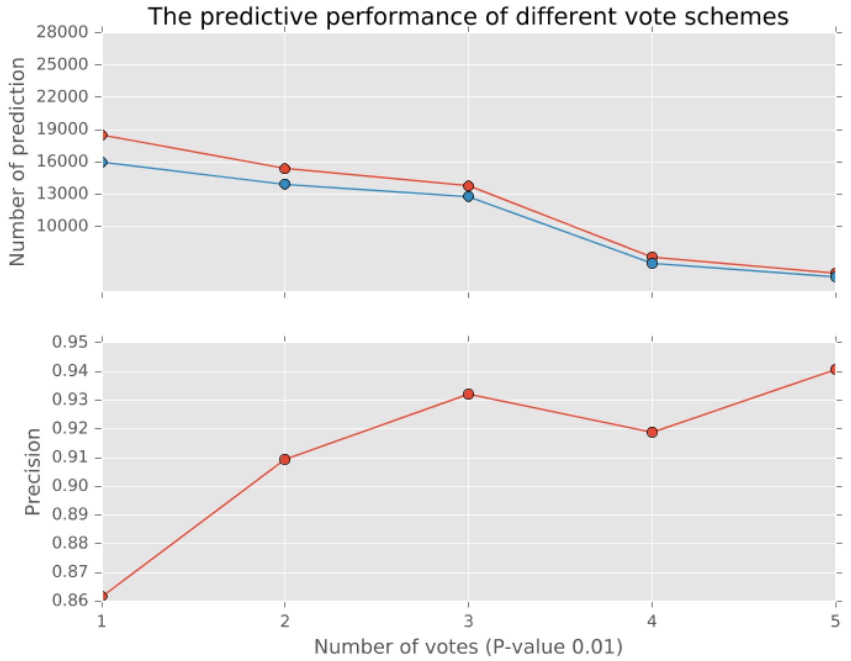


**Figure 4.** The predictive performance of different vote schemes with significant level P-value ≤ 0.01. The upper plot illustrates the total number of positive (in red) and true positive prediction (in blue), and the lower plot is the corresponding precision.

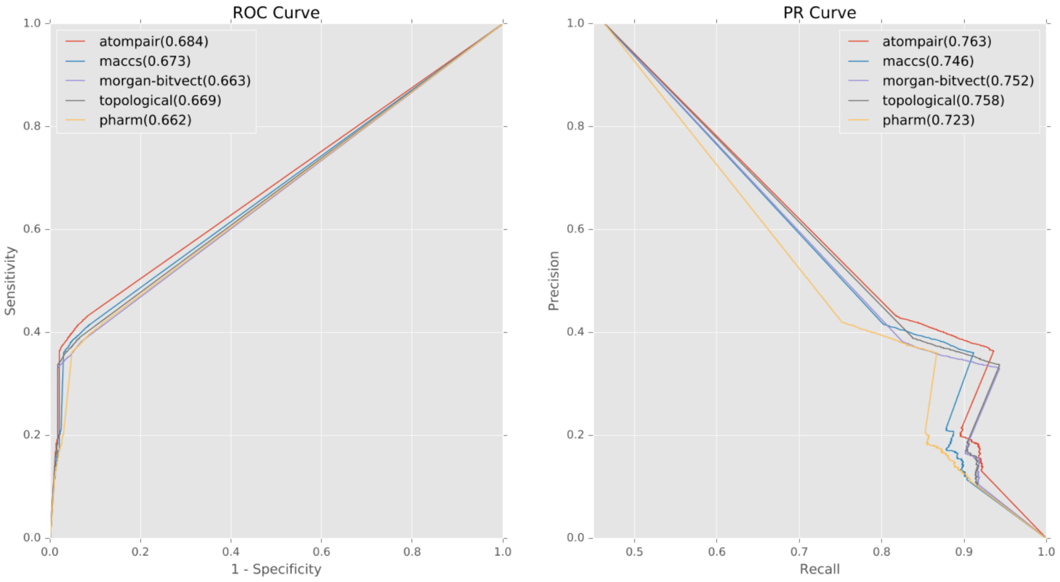


**Figure 5**. The ROC and PR curves of each SEA models on the test set.

Training and testing data sets:

1. 5.csv.tar.bz2: training data set with activity cutoff 10μm.
2. 6.csv.tar.bz2: training data set with activity cutoff 1μm.
3. 7.csv.tar.bz2: training data set with activity cutoff 0.1μm.
4. kinase.csv.tar.bz2: kinase specific training data set with activity cutoff 10μm.
5. test.csv.gz: test data set.
6. kinase test.csv.gz: kinase test data set.
