# Supplementary material for: Improving chemical similarity ensemble approach in target prediction
Source: J Cheminform. 2016 Apr 23;8:20. doi: 10.1186/s13321-016-0130-x (PMC4842302; doi:10.1186/s13321-016-0130-x)
Supplement: Supplementary file 7 — 10.1186/s13321-016-0130-x Figure S1 The average similarity of five fingerprints (Atom pair, Morgan, MACCS, Topological and Pharmacophore, which are implemented in RDKit package [http://rdkit.org/].) and SHED descriptor. The similarity criteria for SHED is the normalized Euclidean distance (see the main manuscript) and for the other five fingerprints are Tanimoto coefficient. Figure S2 Statistical model fits for Morgan based SEA on the random background data set create from ChEMBL 19. Figure S3 Z-score distribution (Morgan fingerprint) of the random background data set created from ChEMBL 19 database. Figure S4 ​The predictive performance of different vote schemes with significant level P-value ≤ 0.01. The upper plot illustrates the total number of positive (in red) and true positive prediction (in blue), and the lower plot is the corresponding precision. [file 13321_2016_130_MOESM7_ESM.pdf]

Additional Figures

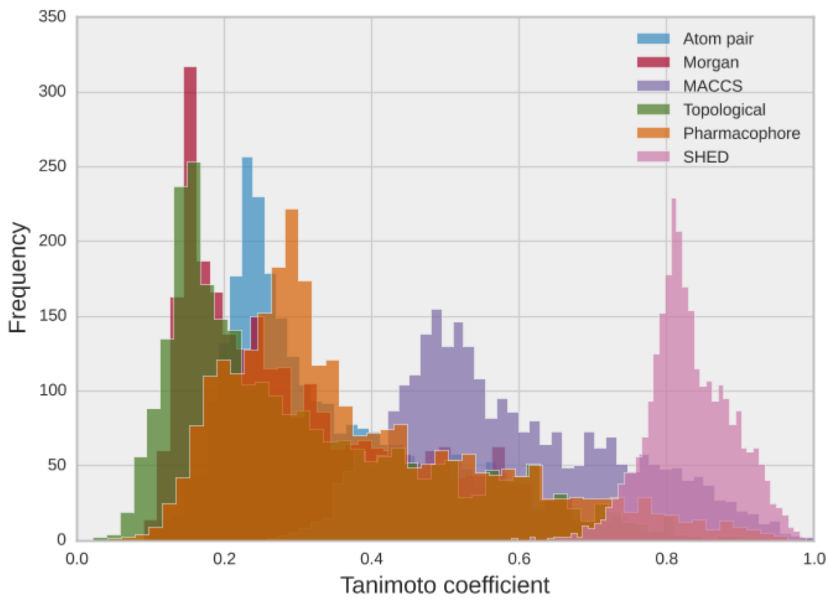

Figure S1. The average similarity of five fingerprints (Atom pair, Morgan, MACCS, Topological and Pharmacophore, which are implemented in RDKit package [http://rdkit.org/].) and SHED descriptor. The similarity criteria for SHED is the normalized Euclidean distance (see the main manuscript) and for the other five fingerprints are Tanimoto coefficient.

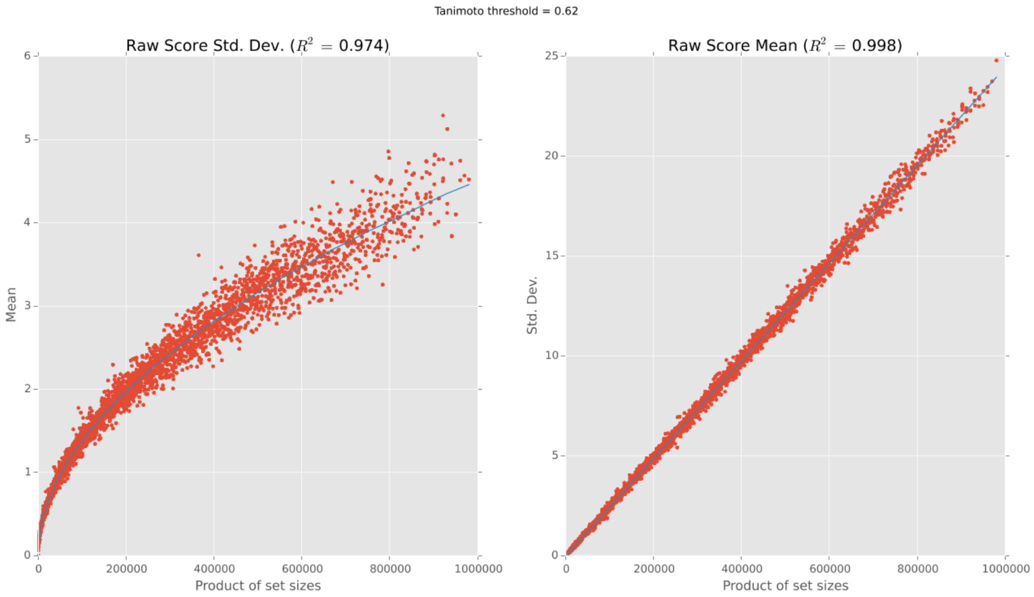

Figure S2. Statistical model fits for Morgan based SEA on the random background data set create from ChEMBL 19.

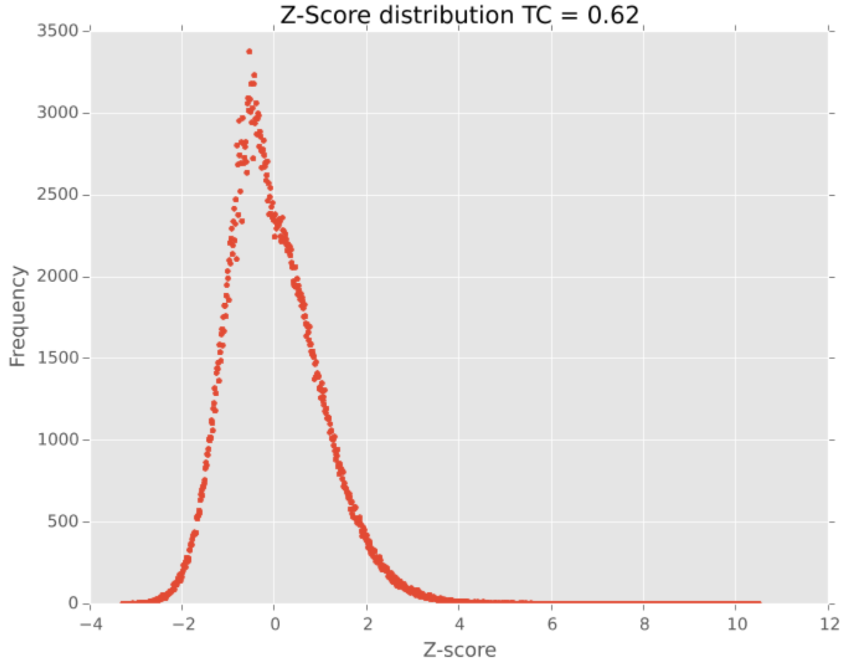

Figure S3. Z-score distribution (Morgan fingerprint) of the random background data set created from ChEMBL 19 database.

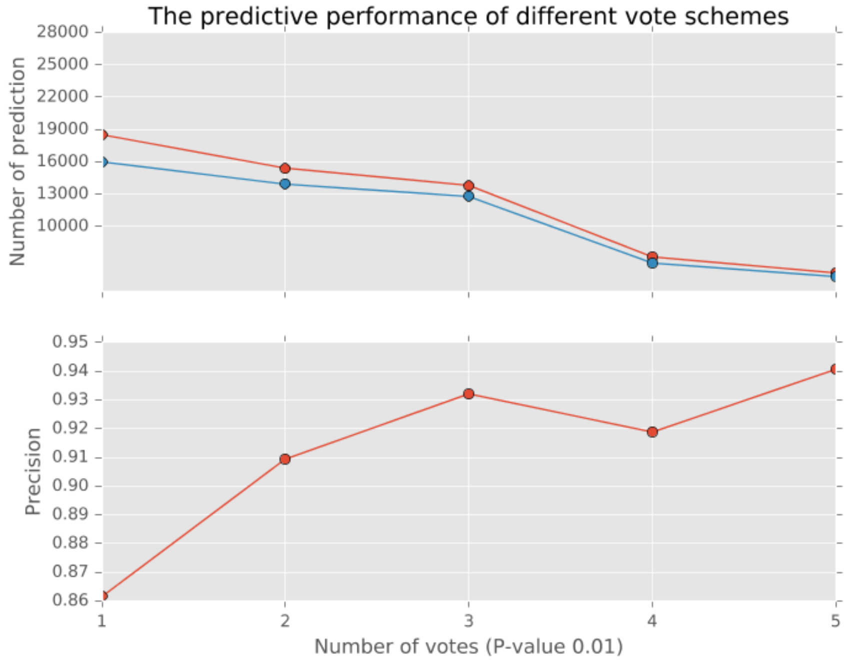

Figure S4. The predictive performance of different vote schemes with significant level P-value ≤ 0.01. The upper plot illustrates the total number of positive (in red) and true positive prediction (in blue), and the lower plot is the corresponding precision.
